# Bis[μ_2_-1,2-bis­(imidazol-1-ylmeth­yl)benzene-κ^2^ *N* ^3^:*N* ^3′^]bis­[dichloridozinc(II)]

**DOI:** 10.1107/S1600536809027044

**Published:** 2009-07-22

**Authors:** Meihong Hu, Shishen Zhang

**Affiliations:** a Department of Biochemistry, College of Technology, Xiaogan University, Xiaogan, Hubei 432000, People’s Republic of China; b Department of Applied Chemistry, Zhejiang Sci-Tech University, Hangzhou 310018, People’s Republic of China

## Abstract

In the crystal structure of the centrosymmetric title compound, [Zn_2_Cl_4_(C_14_H_14_N_4_)_2_], the Zn^II^ atom is coordinated by two N atoms from two 1,2-bis­(imidazol-1-ylmeth­yl)benzene ligands and two Cl atoms to confer a distorted tetra­hedral geometry at the metal center.

## Related literature

For conformationally flexible ligands and their metal complexes, see: Carlucci *et al.* (2004 ▶); Fan *et al.* (2005 ▶); Hennigar *et al.* (1997 ▶). For metal complexes of similar ligands, see: Liu *et al.* (2007 ▶); Moulton & Zaworotko (2001 ▶); Tan *et al.* (2004 ▶).
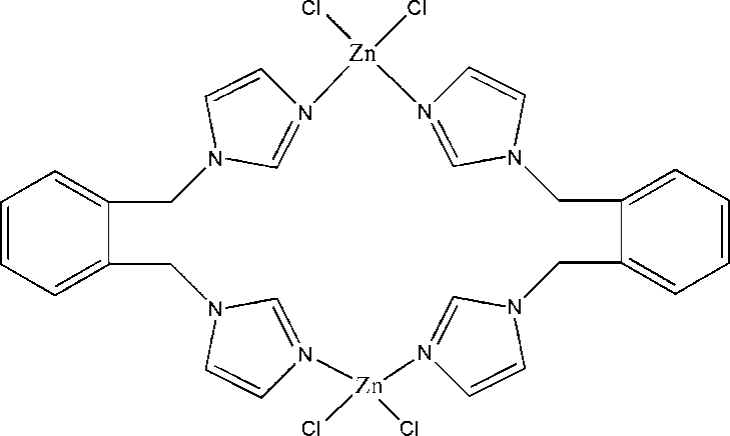


## Experimental

### 

#### Crystal data

[Zn_2_Cl_4_(C_14_H_14_N_4_)_2_]
*M* *_r_* = 749.12
Triclinic, 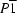

*a* = 8.5502 (12) Å
*b* = 8.7267 (13) Å
*c* = 11.5726 (17) Å
α = 102.824 (2)°
β = 105.720 (2)°
γ = 91.763 (2)°
*V* = 806.6 (2) Å^3^
*Z* = 1
Mo *K*α radiation
μ = 1.85 mm^−1^
*T* = 291 K
0.20 × 0.15 × 0.12 mm

#### Data collection

Bruker SMART APEX CCD diffractometer
Absorption correction: multi-scan (*SADABS*; Bruker, 2000 ▶) *T*_min_ = 0.709, *T*_max_ = 0.809
6327 measured reflections
3130 independent reflections
2912 reflections with *I* > 2σ(*I*)
*R*_int_ = 0.019

#### Refinement

*R*[*F* ^2^ > 2σ(*F* ^2^)] = 0.035
*wR*(*F* ^2^) = 0.084
*S* = 1.09
3130 reflections
190 parameters
H-atom parameters constrained
Δρ_max_ = 0.42 e Å^−3^
Δρ_min_ = −0.27 e Å^−3^

### 

Data collection: *APEX2* (Bruker, 2005 ▶); cell refinement: *SAINT* (Bruker, 2005 ▶); data reduction: *SAINT*; program(s) used to solve structure: *SHELXS97* (Sheldrick, 2008 ▶); program(s) used to refine structure: *SHELXL97* (Sheldrick, 2008 ▶); molecular graphics: *SHELXTL* (Sheldrick, 2008 ▶); software used to prepare material for publication: *SHELXTL*.

## Supplementary Material

Crystal structure: contains datablocks I, global. DOI: 10.1107/S1600536809027044/ng2598sup1.cif

Structure factors: contains datablocks I. DOI: 10.1107/S1600536809027044/ng2598Isup2.hkl

Additional supplementary materials:  crystallographic information; 3D view; checkCIF report

## Figures and Tables

**Table:** 

Zn1—N1	2.000 (2)
Zn1—N2^i^	2.017 (2)
Zn1—Cl1	2.2309 (8)
Zn1—Cl2	2.2428 (8)

**Table:** 

N1—Zn1—N2^i^	108.85 (9)
Cl1—Zn1—Cl2	116.46 (3)

Symmetry code: (i) 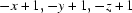.

**Fig. 1.**
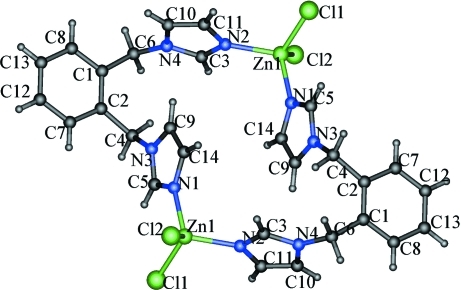
Ellipsoid plot.

**Fig. 2.**
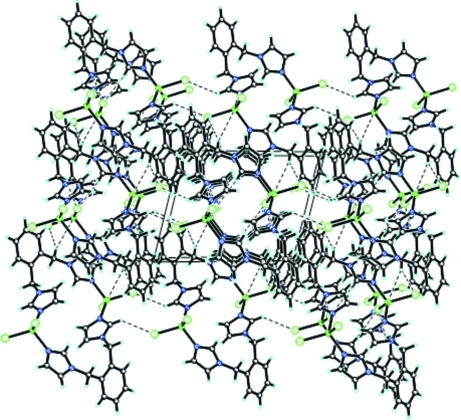
Packing diagram.

## supplementary crystallographic information

### Comment

Conformationally non-rigid ligands, showing varied geometries that often lead to supramolecular isomers, (Moulton et al., 2001; Hennigar et al., 1997) can more easily produce new classes of compounds. 1,2-bis-(imidazol-1-ylmethyl)benzene, as one kind of those ligands, has usually been used to construct a great variety of structurally interesting entities, such as 1-D chain, 2-D and 3-D nets (Liu et al., 2007; Fan et al., 2005; Tan et al., 2004).

The coordination environment of the title compound(I) is illustrated in Fig.1. Single-crystal X-ray diffraction shows that the asymmetric unit contains one Zn crystallographically nonequivalent atom. The Zn atom is coordinated by two N atoms from 1,2-bis-(imidazol-1-ylmethyl)benzene and two Cl atom to give a slightly distorted tetrahedral geometry, forming a 0-D structrue. The crystal packing is stabilized by intermolecular π-π stacking interaction (Fig. 2).

### Experimental

To a test tube (f= 2 cm) containing 3 ml aqueous solution of ZnCl_2_ (0.006 g, 0.05 mmol) was added carefully a layer of THF as a buffer and then 5 ml of methanol solution of 1,2-bis-(imidazol-1-ylmethyl)benzene(0.027 g, 0.1 mmol). The system was allowed to stand for days, during which white crystals were formed in yield of *ca* 45%.

### Refinement

H atoms were positioned geometrically and refined using a riding model, with C—H = 0.93 Å(aromatic) or 0.97 Å(aliphatic) and N—H = 0.86 Å, and with *U*_iso_(H) = 1.2*U_eq_*(C,*N*)

### Crystal data

**Table:**

[Zn_2_Cl_4_(C_14_H_14_N_4_)_2_]	*Z* = 1
*M**_r_* = 749.12	*F*(000) = 380
Triclinic, *P*1	*D*_x_ = 1.542 Mg m^−^^3^
Hall symbol: -P 1	Mo *K*α radiation, λ = 0.71073 Å
*a* = 8.5502 (12) Å	Cell parameters from 3130 reflections
*b* = 8.7267 (13) Å	θ = 1.9–26.0°
*c* = 11.5726 (17) Å	µ = 1.85 mm^−^^1^
α = 102.824 (2)°	*T* = 291 K
β = 105.720 (2)°	Block, white
γ = 91.763 (2)°	0.20 × 0.15 × 0.12 mm
*V* = 806.6 (2) Å^3^	

### Data collection

**Table:**

Bruker SMART APEX CCD diffractometer	3130 independent reflections
Radiation source: fine-focus sealed tube	2912 reflections with *I* > 2σ(*I*)
graphite	*R*_int_ = 0.019
φ and ω scans	θ_max_ = 26.0°, θ_min_ = 1.9°
Absorption correction: multi-scan (*SADABS*; Bruker, 2000)	*h* = −10→10
*T*_min_ = 0.709, *T*_max_ = 0.809	*k* = −10→10
6327 measured reflections	*l* = −13→14

### Refinement

**Table:**

Refinement on *F*^2^	Primary atom site location: structure-invariant direct methods
Least-squares matrix: full	Secondary atom site location: difference Fourier map
*R*[*F*^2^ > 2σ(*F*^2^)] = 0.035	Hydrogen site location: inferred from neighbouring sites
*wR*(*F*^2^) = 0.084	H-atom parameters constrained
*S* = 1.09	*w* = 1/[σ^2^(*F*_o_^2^) + (0.0401*P*)^2^ + 0.3649*P*] where *P* = (*F*_o_^2^ + 2*F*_c_^2^)/3
3130 reflections	(Δ/σ)_max_ = 0.001
190 parameters	Δρ_max_ = 0.42 e Å^−^^3^
0 restraints	Δρ_min_ = −0.27 e Å^−^^3^

### Special details

**Table:**

Geometry. All e.s.d.'s (except the e.s.d. in the dihedral angle between two l.s. planes) are estimated using the full covariance matrix. The cell e.s.d.'s are taken into account individually in the estimation of e.s.d.'s in distances, angles and torsion angles; correlations between e.s.d.'s in cell parameters are only used when they are defined by crystal symmetry. An approximate (isotropic) treatment of cell e.s.d.'s is used for estimating e.s.d.'s involving l.s. planes.
Refinement. Refinement of *F*^2^ against ALL reflections. The weighted *R*-factor *wR* and goodness of fit *S* are based on *F*^2^, conventional *R*-factors *R* are based on *F*, with *F* set to zero for negative *F*^2^. The threshold expression of *F*^2^ > σ(*F*^2^) is used only for calculating *R*-factors(gt) *etc*. and is not relevant to the choice of reflections for refinement. *R*-factors based on *F*^2^ are statistically about twice as large as those based on *F*, and *R*- factors based on ALL data will be even larger.

### Fractional atomic coordinates and isotropic or equivalent isotropic displacement parameters (Å^2^)

**Table:**

	*x*	*y*	*z*	*U*_iso_*/*U*_eq_	
Zn1	0.77170 (3)	0.60926 (3)	0.29452 (3)	0.03231 (11)	
Cl1	0.67332 (9)	0.68391 (9)	0.11848 (6)	0.04696 (19)	
Cl2	1.02202 (9)	0.52603 (9)	0.32757 (7)	0.04806 (19)	
N1	0.6030 (3)	0.4485 (3)	0.2991 (2)	0.0369 (5)	
N2	0.2055 (3)	0.2012 (2)	0.56368 (19)	0.0345 (5)	
N3	0.3737 (3)	0.2956 (2)	0.2378 (2)	0.0348 (5)	
N4	0.1141 (3)	0.0502 (3)	0.37642 (19)	0.0355 (5)	
C1	0.1171 (3)	−0.0585 (3)	0.1590 (2)	0.0354 (6)	
C2	0.2061 (3)	0.0452 (3)	0.1196 (2)	0.0340 (6)	
C3	0.1073 (3)	0.1879 (3)	0.4517 (2)	0.0360 (6)	
H3	0.0415	0.2650	0.4282	0.043*	
C4	0.2099 (3)	0.2226 (3)	0.1610 (3)	0.0397 (6)	
H4A	0.1774	0.2663	0.0890	0.048*	
H4B	0.1321	0.2479	0.2081	0.048*	
C5	0.4628 (3)	0.4045 (3)	0.2123 (2)	0.0372 (6)	
H5	0.4303	0.4445	0.1423	0.045*	
C6	0.0157 (3)	−0.0051 (4)	0.2462 (2)	0.0444 (7)	
H6A	−0.0454	0.0798	0.2214	0.053*	
H6B	−0.0623	−0.0921	0.2393	0.053*	
C7	0.2892 (3)	−0.0154 (4)	0.0350 (3)	0.0430 (7)	
H7	0.3476	0.0535	0.0076	0.052*	
C8	0.1177 (4)	−0.2205 (3)	0.1138 (3)	0.0466 (7)	
H8	0.0601	−0.2909	0.1407	0.056*	
C9	0.4618 (4)	0.2681 (4)	0.3476 (3)	0.0526 (8)	
H9	0.4305	0.1979	0.3888	0.063*	
C10	0.2212 (4)	−0.0315 (3)	0.4443 (3)	0.0493 (7)	
H10	0.2497	−0.1322	0.4167	0.059*	
C11	0.2777 (4)	0.0627 (3)	0.5594 (3)	0.0465 (7)	
H11	0.3536	0.0377	0.6253	0.056*	
C12	0.2873 (4)	−0.1759 (4)	−0.0095 (3)	0.0529 (8)	
H12	0.3440	−0.2143	−0.0663	0.063*	
C13	0.2020 (4)	−0.2781 (4)	0.0301 (3)	0.0536 (8)	
H13	0.2008	−0.3864	0.0006	0.064*	
C14	0.6028 (4)	0.3625 (4)	0.3844 (3)	0.0503 (8)	
H14	0.6865	0.3682	0.4564	0.060*	

### Atomic displacement parameters (Å^2^)

**Table:**

	*U*^11^	*U*^22^	*U*^33^	*U*^12^	*U*^13^	*U*^23^
Zn1	0.03700 (18)	0.02838 (17)	0.02722 (17)	−0.00505 (12)	0.00577 (12)	0.00311 (12)
Cl1	0.0514 (4)	0.0511 (4)	0.0332 (4)	−0.0078 (3)	0.0000 (3)	0.0161 (3)
Cl2	0.0478 (4)	0.0450 (4)	0.0513 (4)	0.0128 (3)	0.0123 (3)	0.0126 (3)
N1	0.0403 (12)	0.0338 (12)	0.0311 (11)	−0.0096 (9)	0.0042 (9)	0.0056 (9)
N2	0.0405 (12)	0.0316 (11)	0.0282 (11)	0.0006 (9)	0.0081 (9)	0.0028 (9)
N3	0.0337 (11)	0.0323 (11)	0.0355 (12)	−0.0045 (9)	0.0059 (9)	0.0078 (9)
N4	0.0420 (12)	0.0327 (12)	0.0289 (11)	−0.0066 (9)	0.0111 (9)	0.0012 (9)
C1	0.0353 (13)	0.0378 (14)	0.0267 (12)	−0.0059 (11)	0.0060 (11)	−0.0004 (10)
C2	0.0315 (12)	0.0367 (14)	0.0289 (13)	0.0000 (11)	0.0036 (10)	0.0044 (11)
C3	0.0409 (14)	0.0315 (13)	0.0331 (14)	0.0015 (11)	0.0096 (11)	0.0041 (11)
C4	0.0315 (13)	0.0351 (14)	0.0472 (16)	−0.0006 (11)	0.0063 (12)	0.0057 (12)
C5	0.0425 (15)	0.0348 (14)	0.0312 (14)	−0.0041 (11)	0.0059 (11)	0.0079 (11)
C6	0.0414 (15)	0.0506 (17)	0.0320 (14)	−0.0166 (13)	0.0093 (12)	−0.0048 (12)
C7	0.0387 (14)	0.0509 (17)	0.0423 (16)	0.0027 (13)	0.0159 (12)	0.0119 (13)
C8	0.0583 (18)	0.0347 (15)	0.0396 (16)	−0.0110 (13)	0.0089 (14)	0.0028 (12)
C9	0.0502 (17)	0.0552 (19)	0.0521 (18)	−0.0132 (14)	0.0030 (14)	0.0290 (15)
C10	0.072 (2)	0.0315 (15)	0.0442 (17)	0.0105 (14)	0.0202 (15)	0.0037 (12)
C11	0.0595 (18)	0.0429 (16)	0.0372 (15)	0.0118 (14)	0.0102 (14)	0.0133 (13)
C12	0.0480 (17)	0.061 (2)	0.0457 (18)	0.0137 (15)	0.0163 (14)	0.0000 (15)
C13	0.065 (2)	0.0349 (16)	0.0481 (18)	0.0098 (14)	0.0053 (15)	−0.0028 (13)
C14	0.0486 (17)	0.0542 (19)	0.0399 (16)	−0.0125 (14)	−0.0049 (13)	0.0187 (14)

### Geometric parameters (Å, °)

**Table:**

Zn1—N1	2.000 (2)	C3—H3	0.9300
Zn1—N2^i^	2.017 (2)	C4—H4A	0.9700
Zn1—Cl1	2.2309 (8)	C4—H4B	0.9700
Zn1—Cl2	2.2428 (8)	C5—H5	0.9300
N1—C5	1.320 (3)	C6—H6A	0.9700
N1—C14	1.366 (4)	C6—H6B	0.9700
N2—C3	1.317 (3)	C7—C12	1.379 (4)
N2—C11	1.371 (4)	C7—H7	0.9300
N2—Zn1^i^	2.017 (2)	C8—C13	1.377 (5)
N3—C5	1.330 (3)	C8—H8	0.9300
N3—C9	1.366 (4)	C9—C14	1.348 (4)
N3—C4	1.475 (3)	C9—H9	0.9300
N4—C3	1.332 (3)	C10—C11	1.350 (4)
N4—C10	1.365 (4)	C10—H10	0.9300
N4—C6	1.476 (3)	C11—H11	0.9300
C1—C2	1.392 (4)	C12—C13	1.363 (5)
C1—C8	1.395 (4)	C12—H12	0.9300
C1—C6	1.509 (4)	C13—H13	0.9300
C2—C7	1.383 (4)	C14—H14	0.9300
C2—C4	1.512 (4)		
			
N1—Zn1—N2^i^	108.85 (9)	N1—C5—N3	111.4 (2)
N1—Zn1—Cl1	106.18 (7)	N1—C5—H5	124.3
N2^i^—Zn1—Cl1	108.10 (7)	N3—C5—H5	124.3
N1—Zn1—Cl2	112.82 (7)	N4—C6—C1	113.2 (2)
N2^i^—Zn1—Cl2	104.18 (7)	N4—C6—H6A	108.9
Cl1—Zn1—Cl2	116.46 (3)	C1—C6—H6A	108.9
C5—N1—C14	105.8 (2)	N4—C6—H6B	108.9
C5—N1—Zn1	123.62 (18)	C1—C6—H6B	108.9
C14—N1—Zn1	130.57 (18)	H6A—C6—H6B	107.7
C3—N2—C11	105.6 (2)	C12—C7—C2	121.4 (3)
C3—N2—Zn1^i^	123.80 (18)	C12—C7—H7	119.3
C11—N2—Zn1^i^	130.53 (19)	C2—C7—H7	119.3
C5—N3—C9	107.0 (2)	C13—C8—C1	121.1 (3)
C5—N3—C4	125.4 (2)	C13—C8—H8	119.4
C9—N3—C4	127.6 (2)	C1—C8—H8	119.4
C3—N4—C10	107.1 (2)	C14—C9—N3	106.7 (2)
C3—N4—C6	125.6 (2)	C14—C9—H9	126.6
C10—N4—C6	127.2 (2)	N3—C9—H9	126.6
C2—C1—C8	118.8 (2)	C11—C10—N4	106.5 (2)
C2—C1—C6	123.4 (2)	C11—C10—H10	126.7
C8—C1—C6	117.8 (3)	N4—C10—H10	126.7
C7—C2—C1	119.0 (2)	C10—C11—N2	109.2 (3)
C7—C2—C4	118.3 (2)	C10—C11—H11	125.4
C1—C2—C4	122.7 (2)	N2—C11—H11	125.4
N2—C3—N4	111.5 (2)	C13—C12—C7	119.8 (3)
N2—C3—H3	124.3	C13—C12—H12	120.1
N4—C3—H3	124.3	C7—C12—H12	120.1
N3—C4—C2	111.8 (2)	C12—C13—C8	119.9 (3)
N3—C4—H4A	109.3	C12—C13—H13	120.1
C2—C4—H4A	109.3	C8—C13—H13	120.1
N3—C4—H4B	109.3	C9—C14—N1	109.1 (3)
C2—C4—H4B	109.3	C9—C14—H14	125.4
H4A—C4—H4B	107.9	N1—C14—H14	125.4

Symmetry codes: (i) −*x*+1, −*y*+1, −*z*+1.
